# Assessment of cellular cobalamin metabolism in Gaucher disease

**DOI:** 10.1186/s12881-020-0947-z

**Published:** 2020-01-13

**Authors:** Suelen Porto Basgalupp, Marina Siebert, Charles Ferreira, Sidney Behringer, Ute Spiekerkoetter, Luciana Hannibal, Ida Vanessa Doederlein Schwartz

**Affiliations:** 1 Postgraduate Program in Medical Sciences, Faculty of Medicine, Universidade Federal do Rio Grande do Sul, Porto Alegre, Brazil; 2 Basic Research and Advanced Investigations in Neurosciences (BRAIN) Laboratory, Experimental Research Center. Hospital de Clínicas de Porto Alegre (HCPA), Porto Alegre, Brazil; 3 Unit of Laboratorial Research, Experimental Research Center, Hospital de Clínicas de Porto Alegre (HCPA), Porto Alegre, Brazil; 4 Postgraduate Program in Health Sciences, Gynecology and Obstetrics (PPGGO), Faculty of Medicine, Universidade Federal do Rio Grande do Sul, Porto Alegre, Brazil; 5 Laboratory of Clinical Biochemistry and Metabolism, Department of General Pediatrics, Adolescent Medicine and Neonatology, Medical Center, Faculty of Medicine, University of Freiburg, Freiburg, Germany; 6 Medical Genetics Service, Hospital de Clínicas de Porto Alegre (HCPA), Porto Alegre, Brazil; 7 Department of Genetics, Universidade Federal do Rio Grande do Sul, Porto Alegre, Brazil

**Keywords:** Gaucher disease, Vitamin B_12_, Cobalamin, Beta-glucosidase, Methylmalonic acid, Homocysteine, Transcobalamin

## Abstract

**Background:**

Gaucher disease (GD) is a lysosomal disorder caused by biallelic pathogenic mutations in the *GBA1* gene that encodes beta-glucosidase (GCase), and more rarely, by a deficiency in the GCase activator, saposin C. Clinically, GD manifests with heterogeneous multiorgan involvement mainly affecting hematological, hepatic and neurological axes. This disorder is divided into three types, based on the absence (type I) or presence and severity (types II and III) of involvement of the central nervous system. At the cellular level, deficiency of GBA1 disturbs lysosomal storage with buildup of glucocerebroside. The consequences of disturbed lysosomal metabolism on biochemical pathways that require lysosomal processing are unknown. Abnormal systemic markers of cobalamin (Cbl, B_12_) metabolism have been reported in patients with GD, suggesting impairments in lysosomal handling of Cbl or in its downstream utilization events.

**Methods:**

Cultured skin fibroblasts from control humans (*n* = 3), from patients with GD types I (*n* = 1), II (*n* = 1) and III (*n* = 1) and an asymptomatic carrier of GD were examined for their GCase enzymatic activity and lysosomal compartment intactness. Control human and GD fibroblasts were cultured in growth medium with and without 500 nM hydroxocobalamin supplementation. Cellular cobalamin status was examined via determination of metabolomic markers in cell lysate (intracellular) and conditioned culture medium (extracellular). The presence of transcobalamin (TC) in whole cell lysates was examined by Western blot.

**Results:**

Cultured skin fibroblasts from GD patients exhibited reduced GCase activity compared to healthy individuals and an asymptomatic carrier of GD, demonstrating a preserved disease phenotype in this cell type. The concentrations of total homocysteine (tHcy), methylmalonic acid (MMA), cysteine (Cys) and methionine (Met) in GD cells were comparable to control levels, except in one patient with GD III. The response of these metabolomic markers to supplementation with hydroxocobalamin (HOCbl) yielded variable results. The content of transcobalamin in whole cell lysates was comparable in control human and GD patients.

**Conclusions:**

Our results indicate that cobalamin transport and cellular processing pathways are overall protected from lysosomal storage damage in GD fibroblasts. Extending these studies to hepatocytes, macrophages and plasma will shed light on cell- and compartment-specific vitamin B_12_ metabolism in Gaucher disease.

## Background

Gaucher disease (GD) is an autosomal recessive inborn error of metabolism caused by deficient activity of glucocerebrosidase (GCase) enzyme due to pathogenic mutations in the *GBA1* gene (OMIM 606463), located on chromosome 1 (1q21) [1]. In rare cases, this disorder can also be caused by a deficiency in the GCase activator, saposin C [2]. GCase catalyzes the conversion of the glycolipid glucocerebroside to ceramide and glucose, and its deficiency leads to the accumulation of this substrate in tissues, especially in the cells of reticuloendothelial system, resulting in dysfunction of different organs such as liver, spleen and bone marrow [1]. GD frequency is estimated to be around 1 in 40,000–60,000 individuals in the general population being more common in the Ashkenazi Jewish affecting 1 in 800 people [3, 4]. This disorder is classified into three main types, based on the absence (type I) or presence and severity (types II and III) of involvement of the central nervous system (CNS) [5]. The diagnosis of GD is performed by measurement of the GCase activity in leukocytes and fibroblasts of individuals with clinical suspicion of the disease. Analysis of the *GBA1* gene is also performed to identify the genotype of the patients. The standard method for variant analysis in GD is full-gene sequencing of *GBA1*. Complementary techniques such as Multiplex Ligation-dependent Probe Amplification (MLPA) can be used to identify deletions or duplications of any region of this gene [6].

The mechanisms of GD pathology are likely multifactorial, with the contribution of genetically unrelated disease modifiers remaining largely unexplored. Abnormalities in systemic markers of cobalamin (Cbl, B_12_) status have been noted in GD patients, raising the question of whether cellular handling of this micronutrient is sensitive to aberrant lysosomal storage [7]. Specifically, studies showed reduced plasma Cbl and elevated holo-transcobalamin (holo-TC) in GD patients [8, 9]. The value of these systemic biomarkers in diagnosing vitamin B_12_ deficiency is limited as they do not measure the status of the two Cbl-dependent enzymes in humans [10, 11]. Cobalamin deficiency inactivates the two Cbl-dependent enzymes methionine synthase and methylmalonyl-CoA mutase, which results in elevation of their substrates, homocysteine (Hcy) and methylmalonic acid (MMA), respectively. Thus, in the absence of folate deficiency (which also leads to elevated tHcy), tHcy and MMA are direct reporters of cellular cobalamin status. Associations between impaired endocytosis and lysosomal metabolism and transient Cbl deficiency have been found in Alzheimer’s disease [12] and in a patient with mutations in the rabenosyn-5 gene [13]. The cellular utilization of vitamin B_12_ requires a functional lysosomal metabolism [10]. Mutations in the lysosomal *cblF* and *cblJ* genes [14–16] responsible for Cbl shuttling from the lysosome into the cytosol, as well as unrelated disturbances of the lysosomal and endocytic pathways [12, 13], lead to functional vitamin B_12_ deficiency and the onset of neurological deterioration. It is currently unknown whether abnormal accumulation of glucocerebroside may affect Cbl transit in and out of the lysosome [7]. Herein, GCase activity, intracellular and extracellular functional markers of Cbl status tHcy and MMA, and expression of the cellular Cbl transporter TC were measured in cultured fibroblasts from healthy human controls and from GD patients. This is the first study to demonstrate an intact Cbl transport and processing axis in Gaucher disease cells. The variable response of cultured GD cells to metabolite reduction upon hydroxocobalamin (HOCbl) supplementation suggest that GD patients presenting with concomitant cobalamin deficiency should be examined on a case-specific basis.

## Methods

### Cell culture

Fibroblasts derived from untreated patients with type I Gaucher’s disease (GM00852), type II (GM00877), type III (GM20272) and one asymptomatic carrier of GD (GM00878) were obtained from the Coriell Institute for Medical Research (Table 1). Healthy fibroblasts were obtained commercially (NHDF), from the Lerner Research Institute, Cleveland Clinic, USA (HFF) [17] or from our clinic from individual without any metabolic diseases (Control-W).

**Table 1:** Genotype and phenotype of healthy and Gaucher disease fibroblasts utilized in this study

Sample	Gender	GD type	Genotype	Remarks
GM00852	Male	I	N370S/84GG	Hepatosplenomegaly; Slowed horizontal saccades.
GM00877	Male	II	L444P/Rec*Nci*I	Hepatosplenomegaly; Strabismus; Trismus.
GM20272	Male	III	L444P/L444P	Hepatosplenomegaly; Slowed horizontal saccades.
GM00878	Female	Carrier of GD	Heterozygous for Rec*Nci*I	Clinically unaffected mother of GM00877.
NHDF	Male	Normal human	No *GBA1* mutation	Healthy dermal fibroblast
HFF	Male	Normal human	No *GBA1* mutation	Healthy dermal fibroblast
Control-W	Female	Normal human	No *GBA1* mutation	Healthy dermal fibroblast

Human controls and GD fibroblasts were cultured in 25 cm^2^ flasks with 5 mL of growth medium (DMEM supplemented with 10% fetal bovine serum (FBS), 1% penicillin-streptomycin in a humidified atmosphere containing 5% CO_2_ at 37 °C) until 80–90% confluency. This culture medium contains no vitamin B_12_, except that present in the 10% FBS as holo-TC (60–70 pM). Trypsin-digestion for cell passages was performed at a ratio of 1:3. Culture medium was exchanged every 2 days, until the beginning of the experiment.

Cell cultures were synchronized such that healthy controls and GD patients were grown simultaneously, beginning on day 1, under the exact same experimental conditions. The experiment was performed in vitamin B_12_- free medium and in medium supplemented with 500 nM HOCbl. Each cell line and condition were grown in triplicate. A sample of culture medium with and without HOCbl supplementation was taken on day 1 of the experiment, and frozen at − 80 °C for further analysis. A total of 42 flasks of cells were maintained in culture at 37 °C for 5 days. Then, conditioned culture medium and cell pellets from each flask were collected and stored at − 80 °C until further analysis.

### β-Glucosidase enzymatic activity assay

#### Preparation of whole cell lysates

Whole cell lysates were prepared freshly on the same day of enzymatic assay testing. The composition of the lysis buffer for the preparation of whole cell lysates was adapted from a published procedure [18] and is part of the diagnostic portfolio of the Metabolic Center Freiburg, Freiburg, Germany. Briefly, cells were lysed in 400 mM Citrate Phosphate buffer supplemented with a protease inhibitor cocktail (Sigma-Aldrich, product Nr. P8340-5ML), Triton X-100 and Sodium-Taurodeoxycholate and centrifuged at 13,000 rpm for 15 min at room temperature (RT). The concentration of proteins in the samples was determined with the Bradford reagent (Bio-Rad, Hercules, CA, USA), using bovine serum albumin (BSA) as standard.

#### β-glucosidase activity in a 96-well microplate assay

A previously reported method for the assessment of β-glucosidase activity [18] was adapted to a 96-well plate assay format for whole cell extract. This assay uses the synthetic substrate 4-methylumbelliferyl-β-D-glucopyranoside (4MUβ-Glucopyranoside, Sigma-Aldrich). Briefly, cell lysates containing 0.1 micrograms of protein were transferred to a 96-well microplate, each sample by triplicate. The reaction buffer consisted of 400 mM citrate phosphate buffer (pH 5.2), 14.3 mM sodium taurodeoxycholate and 6 mM 4MUβ-Glucopyranoside (90 μl). The samples were incubated in reaction buffer for 18 h at 37 °C. The reaction was stopped by the addition of 110 μl of Glycine buffer (0.5 M, pH 10.4). The amount of fluorescent product formed was measured with an Infinite® 200 PRO plate reader (Tecan, Life Sciences) set up with fluorescence excitation at 355 nm and fluorescence emission at 460 nm. As a control of quality of the specimen, we measured α-glucosidase enzymatic activity assay using the substrate 4-methylumbelliferyl-α-D-glucopyranoside (Sigma-Aldrich). This lysosomal protein is expected to be intact in GD cell lines. The assay conditions were exactly as described herein for β-glucosidase activity, except that 4-methylumbelliferyl-α-D-glucopyranoside was used as the substrate.

### Content of transcobalamin in whole cell lysates

The content of TC in control human and GD fibroblasts was examined by Western blot, with whole cell extracts prepared under near-native conditions as described above for the β-glucosidase activity measurement. From that extract, 15–20 μl (30 micrograms of total protein) was loaded on an SDS-PAGE for Western blotting. The primary antibody (rabbit anti-human TC; 189,871; Abcam, Cambridge, United Kingdom) was used at a dilution of 1:500 and the secondary antibody at 1:1000 (polyclonal goat anti-rabbit) as reported previously [19]. Semi-quantitative analysis of western blots was performed with Image J software freely available from the National Institutes of Health, USA [20].

### Analysis of intracellular and extracellular tHcy, Cys and met by LC-ESI-MS/MS

#### Extraction of aminothiols from cultured cells

Cultured fibroblasts were harvested by trypsinization, washed with PBS, and frozen at − 80 °C until further analysis. After thawing the cell pellets, 0.1 ml of 20 mM dithiothreitol (DTT) was added. Lysis was performed by freeze-thawing of cells by alternating between dry-ice and RT, three times. An aliquot of 10 μl of lysate was separated and stored at − 80 °C for further measurement of concentration of proteins with the Bradford reagent (Bio-Rad, Hercules, CA, USA). Then, 0.1 ml 10% trifluoroacetic acid (TFA) was added to precipitate proteins. The extracts were incubated at RT for 15 min, and then centrifuged at 13,000 rpm for 15 min at RT. An aliquot of 3.16 μl of aminothiol-containing supernatant (intracellular aminothiols) or culture medium (extracellular aminothiols) was transferred into a clean Eppendorf tube. A 20 μl aliquot of Internal Standard (Stable isotopically labelled homocysteine, cysteine and methionine) was then added to each tube followed by 20 μl of 0.5 M DTT solution and 100 μl of methanol with 0.1% formic acid. The mixtures were vortexed for 10 s at a medium speed and incubated for 20 min at RT. After that, the samples were centrifuged at 10,000 g for 5 min. An aliquot of 60 μl of supernatant was transferred into high performance liquid chromatography (HPLC) vials and 10 μl of each sample was injected into the HPLC equipment. Two commercial controls having known amounts of Hcy, cysteine (Cys) and methionine (Met) were used as a quality control (“Special Assays in Serum” and “control amino acids”, MCA Laboratory, Queen Beatrix Hospital in 7101 BN Winterswijk, The Netherlands). The concentration of aminothiols was determined with respect to a calibration curve and the addition of stable isotopically labelled internal standards [21]. Briefly, calibrators were prepared by preparing a master mix containing 100 μl of 0.5 M DTT, 100 μl of Hcy (998.6 μM), 150 μl of Met (998.5 μM), 300 μl of Cys (1007 μM), 150 μl of methionine sulfoxide (1000 μM) and 200 μl of H_2_O (Calibrator 1). A serial dilution was performed by pipetting 200 μl of Calibrator 1 into an Eppendorf tube containing 200 μl H_2_O (Calibrator 2), up to calibrator 7. The calibrators were vortexed. Aliquots of 3.16 μl of each calibrator were pipetted into HPLC vials and 20 μl of internal standard solution (D_4_-Homocysteine (20 μM), D_4_-Methionine (60 μM), ^13^C_3_-Cysteine (102.3 μM) in H_2_O) was added to each vial followed by 20 μl of 0.5 M DTT solution and 100 μl of methanol with 0.1% formic acid. After vortexing, 10 μl of the samples was injected into the liquid chromatography electrospray ionization tandem mass spectrometry (LC-ESI-MS/MS) machine (QTrap 6500+, Sciex). Values of intracellular and extracellular tHcy-, Cys- and Met- concentration were normalized to total protein concentration.

### Analysis of intracellular and extracellular methylmalonic acid (MMA) by LC-ESI-MS/MS

Methylmalonic acid was determined based on a previously published method with modifications [22]. Cultured fibroblasts were lysed using the same protocol described for aminothiols. For sample preparation, an aliquot of 100 μl of cell lysate (intracellular MMA) and conditioned culture medium (extracellular MMA) of each healthy human control and Gaucher fibroblasts was pipetted into a clean microcentrifuge filter (Amicon, 30 kDa MW cut-off, Merck Millipore) tube and 100 μl of 0.8 μM D_3−_MMA (CDN isotopes) Internal Standard solution was added to each tube. The samples were vortexed for 10 s and centrifuged at 14,000 g for 30 min at 10 °C. After centrifugation, 100 μl of the filtrate was transferred into HPLC vials and acidified with 10 μl of 4% formic acid. Then, 10 μl of the sample was injected into the LC-ESI-MS/MS system (QTrap 6500+, Sciex). Two commercial controls (Special Assays in Serum, MCA Laboratory, Queen Beatrix Hospital in 7101 BN Winterswijk, The Netherlands that have known concentrations of MMA were used for analysis. Quantification of MMA was performed by use of a calibration curve. Briefly, 100 μl of MMA standards (0.1, 0.25, 0.5, 0.75, and 1.0 μM) were pipetted into Eppendorf tubes and 100 μl of 0.8 μM D_3_-MMA internal standard solution was added to each tube. After vortexing, a 100 μl aliquot was transferred into HPLC vials and acidified with 10 μl of 4% formic acid. Then, 10 μl of each sample was injected into the LC-ESI-MS/MS system. Stock solutions of MMA (Sigma Aldrich) and D_3_-MMA were prepared in deionized water and kept at − 20 °C. Values of intracellular and extracellular MMA were normalized to total protein concentration of the cell lysates.

### Statistical analysis

Regarding the data processing, database double entry, review and analysis were performed using the SPSS, version 18.0 [SPSS Inc. Released 2009. PASW Statistics for Windows, Version 18.0. Chicago: SPSS Inc.].

Quantitative data was expressed by median and 95% Confidence Interval [95%CI]. To compare medians between groups (control versus GD) or cobalamin supplementation (with versus without the presence of HOCbl supplementation), the Mann-Whitney test for independent samples was used to perform comparisons. For assessing possible interactions between both factors, the Kruskal-Wallis test for independent samples, with Dunn post hoc test, was applied. The level of significance was set at 5% for all analysis.

## Results

The genotypes and phenotypes of healthy human controls, GD patients and asymptomatic carrier of GD are displayed in Table 1. The skin fibroblasts of GD patients used in this study have been characterized extensively in previous work at the enzymatic, organelle and metabolic levels [23–29]. Our study focuses on markers of vitamin B_12_ status, which are examined for the first time.

### α- and β-glucosidase activities of fibroblasts derived from healthy human controls, Gaucher disease patients and an asymptomatic carrier of GD

**Fig. 1:**
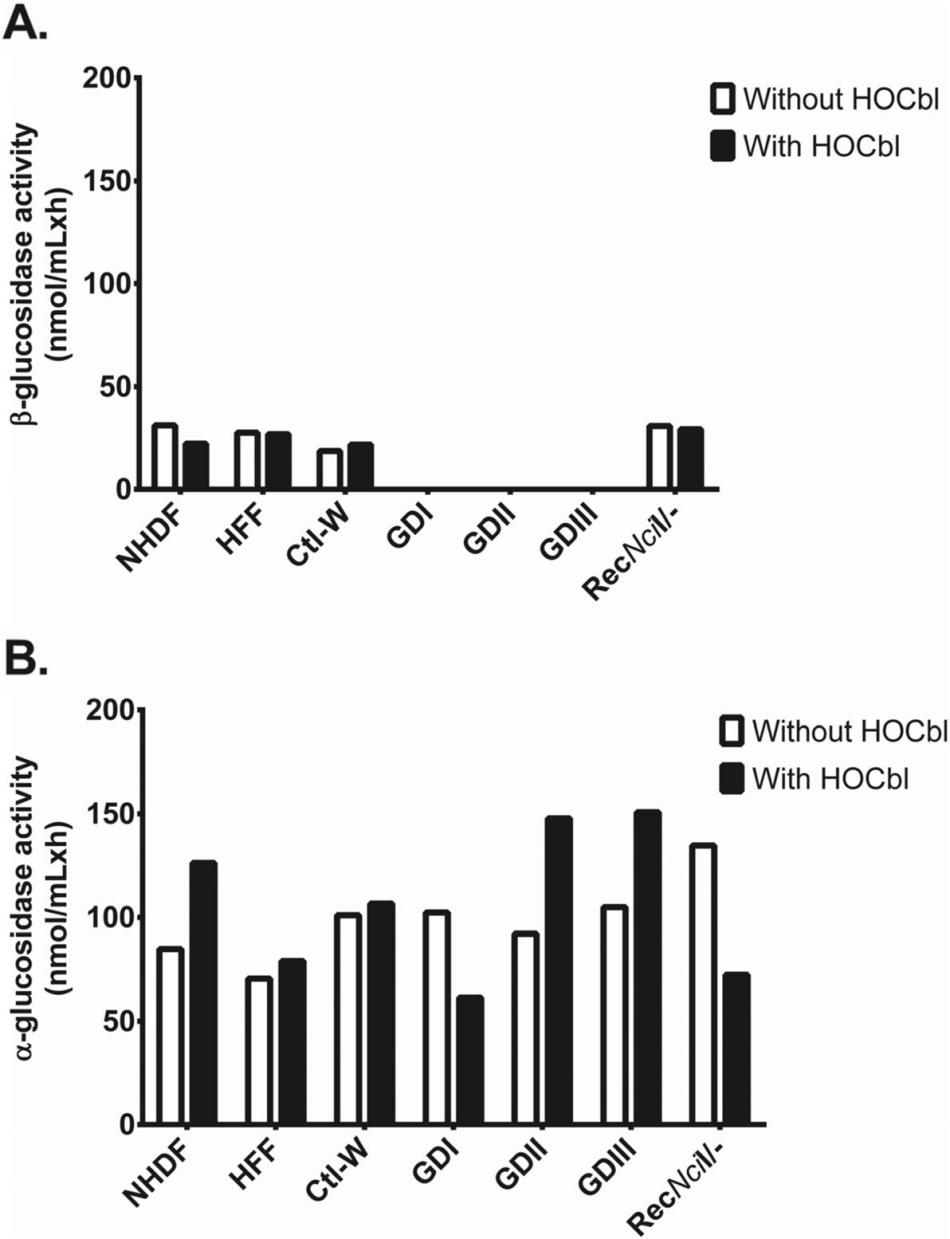
α- and β-glucosidase activity in healthy human controls, Gaucher disease patients and an asymptomatic carrier of GD. **a** Gaucher patients (*n* = 3) had no detectable activity of β-glucosidase (nmol/mLxh), whereas the asymptomatic carrier of GD (*n* = 1) exhibited β-glucosidase activity comparable to those of human controls (*n* = 3); **b** All examined subjects presented comparable α-glucosidase activity (nmol/mLxh), suggesting preserved activity of lysosomal components not associated with the *GBA1* mutation. Legend: without HOCbl – culture medium without hydroxocobalamin; with HOCbl – culture medium with hydroxocobalamin, GD – Gaucher disease, Rec*Nci*I/− − asymptomatic carrier of GD

GCase activity was measured in whole cell extracts of control human and GD fibroblasts. GCase activities in the GD (Types I, II and III) cell lines were consistently lower than those in control and asymptomatic carrier of GD cells (Fig. 1 and Table 2). The enzymatic assay confirmed that GD cells homozygous or compound heterozygous for *GBA1* had no detectable β-glucosidase activity (Fig. 1a). Additionally, the results demonstrate the deficiency of GCase activities in GD fibroblasts and that heterozygous for *GBA1* had GCase activity almost as high as control cells, suggesting a compensatory mechanism provided by the wild type *GBA1* allele. The GCase activity was not markedly affected by the presence of HOCbl supplementation in the culture medium (black bars).

**Table 2:** β and α-glucosidase activities of human controls and Gaucher disease fibroblasts

Cell lines	β-glucosidase activity (nmol/mLxh)		α-glucosidase activity (nmol/mLxh)	
	Without HOCbl	With HOCbl	Without HOCbl	With HOCbl
NHDF	31.2	22.2	84.8	126.3
HFF	27.6	26.9	70.6	79.1
Control-W	18.7	21.8	101.0	106.6
GM00852 (GD I)	ND	ND	102.3	61.4
GM00877 (GD II)	ND	ND	92.1	147.8
GM20272 (GD III)	ND	ND	104.9	150.7
GM00878 (Rec*Nci*I/−)	30.9	29.3	134.7	72.4

Legend: without HOCbl – culture medium without hydroxocobalamin; with HOCbl – culture medium with hydroxocobalamin; *ND* not detectable; *NHDF* Normal Human Dermal Fibroblast; *HFF* Human foreskin fibroblast; *Control-W* Control W fibroblast; *GD I* Gaucher disease type I; *GD II* Gaucher disease type II; *GD III* Gaucher disease type III; *RecNciI/* Asymptomatic carrier of GD

To evaluate the quality of the specimen, the lysosomal α-glucosidase activity was measured and revealed that healthy individuals and GD fibroblasts exhibit an intact α-glucosidase activity (Fig. 1b and Table 2). These results exclude the occurrence of unwanted damage of relevant enzymatic lysosomal components during sample preparation.

### Content of transcobalamin, the cellular transporter of cobalamin

**Fig. 2:**
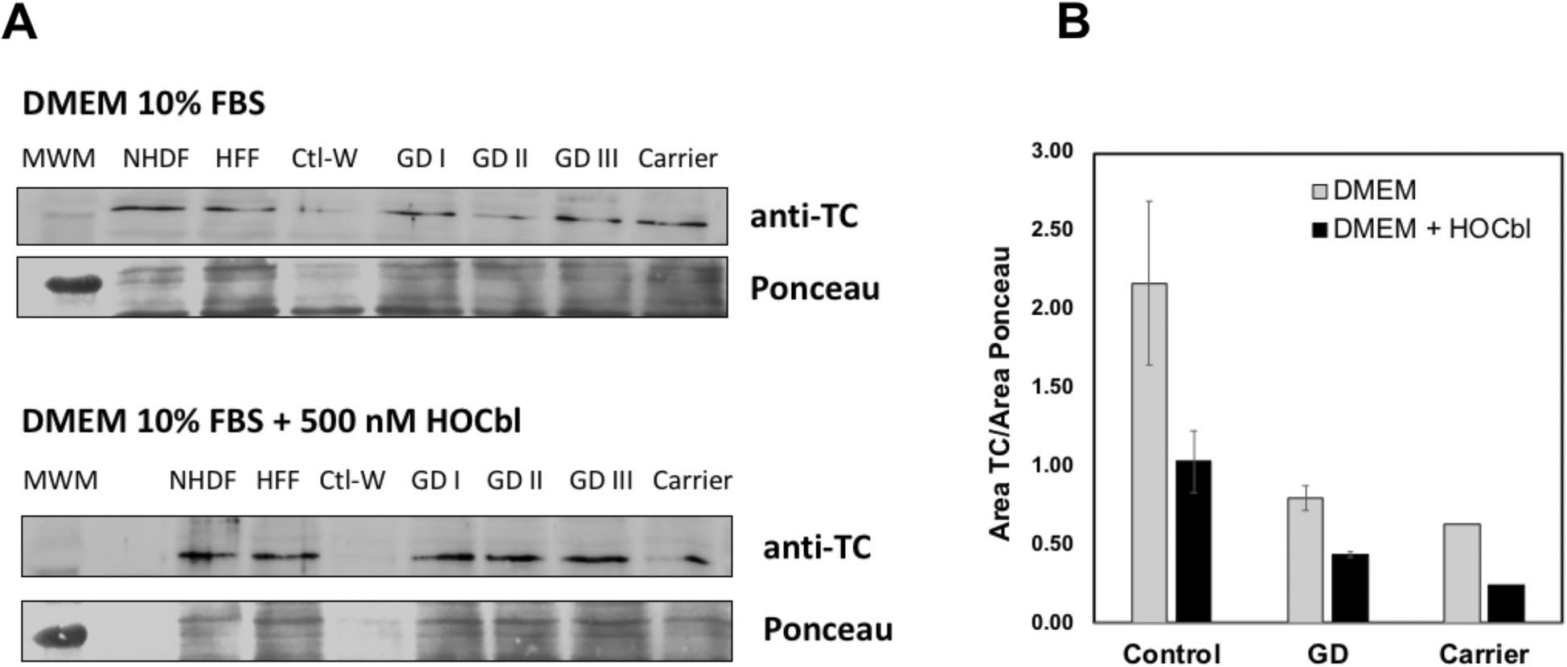
Content of transcobalamin in healthy human controls, Gaucher disease patients and an asymptomatic carrier of GD. **a** Whole cell lysates (30 μg) of human control and GD fibroblasts were examined for intracellular content of transcobalamin, with and without HOCbl supplementation. Under our experimental conditions, no differences were identified between control and GD cells, suggesting normal expression of transport protein transcobalamin. Left panel: Western blot results obtained by probing whole cell lysates with anti-TC (rabbit anti-human, dilution 1:500) and secondary goat anti-rabbit IgG-HRP (1:1000) antibody. Right panels: Ponceau staining of nitrocellulose membrane after semi-dry blot transfer as protein loading control. **b** Semi-quantitative analysis of TC content shown in panel (**a**) shows slightly reduced TC content in GD cells compared to healthy human controls, albeit without statistical significance. Values shown are mean normalized areas plus minus standard error of the mean

Because cobalamin reaches all cells in the body bound to transporter protein TC, and once inside the cell, release of cobalamin for downstream use is preceded by lysosomal degradation of TC, we examined whether abnormal lysosomal storage brought about by mutations in *GBA1* affect the TC in cells. Expression of TC has been demonstrated in a variety of human cell types [19, 30–37], including fibroblasts [38, 39]. Western blot analysis of whole cell lysates indicated comparable content of TC in healthy individuals and GD fibroblasts (Fig. 2a). A slightly decreased content of TC was documented in GD cells but this difference was not statistically significant (Fig. 2b). Given the widespread expression of TC in a number of different cell types and the results obtained herein, the previously reported abnormalities in plasma holo-TC in GD patients [9] do not seem to arise from abnormal biosynthesis/turnover of this protein in GD cells.

### Intracellular tHcy, Cys, met and MMA in Gaucher disease and response to supplementation with hydroxocobalamin

Aiming to examine cellular Cbl status in GD (*n* = 3) and healthy human controls (*n* = 3), we examined the intracellular concentration of marker metabolites of Cbl status, tHcy and MMA, as well as markers of the methionine cycle, Met, and of the trans-sulfuration pathway, Cys.

The intracellular concentrations of tHcy, MMA and Cys were comparable in healthy human controls, GD patients and the asymptomatic carrier. A group effect was observed in Met, since GD participants showed increased median compared to control group (Mann-Whitney test, *p* = 0.015; Table 3). Additionally, no group, cobalamin supplementation or interaction effect was observed in all other assessed variables intracellularly (Mann-Whitney test or Kruskal-Wallis test with Dunn post hoc, *p* > 0.05 for all, Table 3).

**Table 3:** Intracellular levels of tHcy, Cys, Met and MMA in Gaucher disease and healthy human fibroblasts in the absence and in the presence of HOCbl

Variable	Healthy human fibroblasts		GD fibroblasts		**p-value		
	Without HOCbl (*n*=3)	With HOCbl (*n* = 3)	Without HOCbl (*n* = 3)*	With HOCbl (*n* = 3)*	Group	Cobalamin supplementation	Interaction
tHcy (nmol/mg) range	0.21[0.04–0.45] 0.19–0.34	0.17[0.12–0.22] 0.15–0.19	0.26[−0.12–0.77] 0.19–0.53	0.25[0.10–0.40] 0.19–0.31	0.180	0.180	0.154
Cys (nmol/mg) range	34.2[10.21–53.57] 22.24–39.23	36.26[6.80–64.97] 23.99–47.40	31.67[−10.99–87.88] 22.82–60.85	27.93[−22.75–102.04] 22.53–68.48	1.000	0.818	0.954
Met (nmol/mg) range	27.72[15.90–40.19] 23.33–33.09	32.49[20.65–40.67] 26.04–33.45	39.73[22.03–55.31] 31.50–44.77	38.59[23.84–59.61] 36.62–49.96	**0.015**	0.589	0.103
MMA (nmol/mg) range	0.01[0.01–0.02] 0.01–0.02	0.01[0.01–0.03] 0.01–0.02	0.02[−0.01–0.05] 0.01–0.03	0.02[− 0.02–0.06] 0.01–0.04	0.699	0.818	0.965

Data expressed as Median [95% Confidence Interval], range. *n* = 3 for each group. Legend: without HOCbl – culture medium without hydroxocobalamin; with HOCbl – culture medium with hydroxocobalamin; *GD* Gaucher Disease. *p* – statistical significance. **Mann-Whitney test (factors: group – healthy human and GD patients, cobalamin supplementation) or Kruskal-Wallis test with Dunn post hoc (factors: group – healthy human control and GD; cobalamin supplementation; interaction) for independent samples. Significance set at 5% for all analysis. *GD types I (*n* = 1); II (*n* = 1) and III (*n* = 1). Statistically significant findings are highlighted in bold font

### Extracellular tHcy, Cys, Met and MMA in Gaucher disease and response to supplementation with hydroxocobalamin

Cells maintain healthy intracellular concentrations of tHcy and MMA partly through the export of these metabolites into the extracellular milieu (to circulation in whole organisms, and to conditioned culture medium in cultured cells). The levels of tHcy, Cys, Met and MMA were comparable between control (*n* = 3) and GD (*n* = 3) conditioned medium (Table 4). The concentration of tHcy was significantly decreased after supplementation with HOCbl in healthy human controls and GD fibroblasts (Mann-Whitney test, *p* = 0.041), with no effect of this supplementation on Cys, Met and MMA (Mann-Whitney test, *p* > 0.05). Furthermore, no group effect or interaction was observed among all other assessed variables extracellularly (Mann-Whitney test or Kruskal-Wallis test with Dunn post hoc, *p* > 0.05 for all, Table 4).

**Table 4:** Extracellular levels of tHcy, Cys, Met and MMA in Gaucher disease and healthy human fibroblasts in the absence and in the presence of HOCbl

Variable	Healthy human fibroblasts		GD fibroblasts		**p-value		
	Without HOCbl (*n* = 3)	With HOCbl (*n* = 3)	Without HOCbl (*n* = 3)*	With HOCbl (*n* = 3)*	Group	Cobalamin supplementation	Interaction
tHcy (nmol/mg) range	3.74[3.01–4.45] 3.44–4.02	2.15[0.22–3.44] 1.08–2.25	3.73[1.87–5.27] 2.82–4.16	1.93[− 0.88–5.92] 1.54–4.08	0.699	**0.041**	0.187
Cys (nmol/mg) range	142.88[5.70–280.12] 87.69–198.16	159.23[26.33–271.79] 95.36–192.59	284.72[−115.81–648.73] 104.26–410.40	266.75[−192.00–807.51] 130.24–526.28	0.132	1.000	0.459
Met (nmol/mg) range	116.05[31.86–212.08] 89.02–160.84	126.66[36.86–214.55] 89.47–160.98	156.44[−53.85–368.93] 73.00–243.18	148.51[−74.35–424.63] 90.70–286.20	0.589	0.699	0.887
MMA (nmol/mg) range	0.14[0.01–0.26] 0.08–0.18	0.01[−0.09–0.46] 0.06–0.27	0.23[0.01–0.37] 0.10–0.23	0.23[−0.01–0.49] 0.14–0.34	0.310	0.394	0.536

Data expressed as Median [95% Confidence Interval]. Legend: *without HOCbl* culture medium without hydroxocobalamin; *with HOCbl* culture medium with hydroxocobalamin; *GD* Gaucher Disease. *p* – statistical significance. **Mann-Whitney test (factors: group – healthy human and GD patients, cobalamin supplementation) or Kruskal-Wallis test with Dunn post hoc (factors: group – healthy human control and GD; cobalamin supplementation; interaction) for independent samples. Significance set at 5% for all analysis. *GD types I (*n* = 1); II (*n* = 1) and III (*n* = 1). Statistically significant findings are highlighted in bold font

## Discussion

The primary objective of this work was to examine cobalamin metabolism in GD cells. The investigation of cobalamin metabolism in GD was partly motivated by previous reports indicating a high incidence of low serum vitamin B_12_ in untreated GD patients [8], increased circulating levels of transcobalamin II (TCII) in GD patients [9], and slightly increased Hcy and MMA in polyneuronopathic GD type I patients compared to non-neuronopathic patients [40]. In the latter study, it is worth mentioning that the tHcy and MMA elevation documented in polyneuropathic GD type I patients versus those without signs of polyneuropathy was mild, within the reference range considered normal.

Cultured human fibroblasts are an invaluable resource in the diagnosis of metabolic diseases. Skin fibroblasts from Gaucher patients exhibit a severe deficiency of GCase activity [41], which facilitates the investigation of metabolic abnormalities in vitro. The specific subset of skin fibroblasts from GD patients employed herein have been profusely characterized in previous work at the cellular, organelle and enzymatic levels [23–29]. Our results confirmed the deficiency activity of GCase in GD fibroblasts types I, II and III, and showed that an asymptomatic carrier of GD had enzymatic activity similar to control human cells. This in vitro result is consistent with the asymptomatic phenotype of patients heterozygous for *GBA1*, and suggest that one functional allele is sufficient to compensate GCase activity.

Skin fibroblasts have been the most widely utilized cell type to investigate disorders of vitamin B_12_ metabolism over several decades [42, 43]. Therefore, our results of marker metabolites of vitamin B_12_ status and holo-TC in this cell type can be directly compared to numerous studies of canonical inborn errors of vitamin B_12_ metabolism, all of which have been performed in skin fibroblasts. The assessment of cobalamin status should include preferentially a combination of functional markers (tHcy, MMA) and systemic markers (plasma B_12_ and holo-TC) [44–47]. Intracellular tHcy was slightly increased in GD cells, but the difference with respect to control did not hold statistical significance. We found comparable levels of intracellular and extracellular Cys and Met in control and GD cells, suggesting a functional methionine cycle and steady state of Cys concentration. Intracellular MMA was elevated in GD III cells and did not respond to supplementation with hydroxocobalamin, but this was not observed in GD I and II or in an asymptomatic carrier of GD. Extracellular tHcy, Met and Cys were comparable in control human and GD patients, with the exception of GD III exhibiting elevated Met and Cys compared to control, none of which responded to hydroxocobalamin supplementation. Extracellular MMA was only elevated in GD III and did not respond to HOCbl supplementation, whereas in the other GD cells levels of this metabolite were comparable to control human fibroblasts. Regardless of exogenous HOCbl supplementation all GD cells exhibited slightly reduced content of TC compared to healthy human controls, but this difference holds no statistical significance. This finding is in line with our metabolomic examination highlighting no indication of disrupted cobalamin metabolism. The content of holo-TC in cells treated with HOCbl showed a trend similar to the reduction of marker metabolites Hcy and MMA. Canonical inborn errors of cobalamin involving the lysosome, such as cblF and cblJ [14, 16, 48–50] and other lysosomal defects that impair cobalamin metabolism secondarily such as mutations in the rabenosyn-5 gene or defective lysosomal acidification in Alzheimer’s disease [12, 13], feature one or all of the classical marker metabolite trends, namely, elevated Hcy and MMA and reduced Met in cells and/or plasma. Our experimental results suggest an overall preserved metabolism of cobalamin in GD cells. While we did not find evidence of disturbed cobalamin metabolism in skin fibroblasts, a possibility exists that abnormal lysosomal storage in other cell-types alters their cobalamin transporters and metabolism in the lysosome, including: (a) the retention and efflux of cobalamin in the organelle over time, and (b) the distribution and interactions of lysosomal cobalamin transporters LMBRD1 and ABCD4. This distinct possibility awaits further investigation.

## Conclusion

The comparable levels of marker metabolites Hcy, MMA and Met and content of holo-TC in cells from healthy controls and GD patients suggest that cobalamin transport and processing pathways are overall preserved in GD cells, that is, insensitive to the lack of β-glucosidase activity that leads to abnormal lysosomal storage. Based on previous literature and our study, we suggest that GD patients who present with concomitant cobalamin deficiency should be examined on a case-specific manner with respect to potential use of and response to cobalamin.

### Limitations of this study

Our studies were performed with skin fibroblasts. Skin fibroblasts have been used extensively to investigate various aspects of GD, including β-glucosidase activity [23–29], and is the cell type of choice for the study of inborn errors of cobalamin metabolism. However, clinical presentations in patients indicate that the most relevant cell types in the pathogenesis of GD are macrophages and hepatocytes. It would be of great interest to recreate our studies in these cell types, shall they become readily available for research purposes. Another limitation of our study is the low number of GD cell lines examined with respect of the variability of clinical manifestations of patients with GD I, GD II and GD III. Further research with a greater number of samples may reveal so far unrecognized associations between GD and vitamin B_12_ metabolism. Because a distinct correlation between serum markers of Cbl (from published work) [9, 40, 44] and cellular status of the micronutrient is not always observed, it would be of great value to investigate markers of vitamin B_12_ status in paired plasma and cell samples from the same GD patients and in healthy human controls.

## Data Availability

The datasets used and analyzed during the current study are available from the corresponding author on reasonable request.

## Abbreviations

4MUβ-Glucopyranoside: 4-methylumbelliferyl-β-D-glucopyranoside
B_12_: Vitamin B_12_
BSA: Bovine serum albumin
Cbl: Cobalamin
CNS: Central nervous system
Cys: Cysteine
DDT: Dithiothreitol
FBS: Fetal bovine serum
GCase: Beta-glucosidase enzyme
GD: Gaucher disease
HOCbl: Hydroxocobalamin
Holo-TC: Holo-transcobalamin
HPLC: High performance liquid chromatography
LC-ESI-MS/MS: Liquid chromatography electrospray ionization tandem mass spectrometry
Met: Methionine
MLPA: Multiplex Ligation-dependent Probe Amplification
MMA: Methylmalonic acid
PBD: Phosphate-buffered saline
RT: Room temperature
TC: Transcobalamin
TFA: Trifluoroacetic acid
tHcy: Total homocysteine
